# Hearing, touching, and multisensory integration during mate choice

**DOI:** 10.3389/fncir.2022.943888

**Published:** 2022-09-20

**Authors:** Constanze Lenschow, Ana Rita P. Mendes, Susana Q. Lima

**Affiliations:** Champalimaud Foundation, Champalimaud Research, Neuroscience Program, Lisbon, Portugal

**Keywords:** somatosensation, audition, multisensory integration, mate choice, neural circuit

## Abstract

Mate choice is a potent generator of diversity and a fundamental pillar for sexual selection and evolution. Mate choice is a multistage affair, where complex sensory information and elaborate actions are used to identify, scrutinize, and evaluate potential mating partners. While widely accepted that communication during mate assessment relies on multimodal cues, most studies investigating the mechanisms controlling this fundamental behavior have restricted their focus to the dominant sensory modality used by the species under examination, such as vision in humans and smell in rodents. However, despite their undeniable importance for the initial recognition, attraction, and approach towards a potential mate, other modalities gain relevance as the interaction progresses, amongst which are touch and audition. In this review, we will: (1) focus on recent findings of how touch and audition can contribute to the evaluation and choice of mating partners, and (2) outline our current knowledge regarding the neuronal circuits processing touch and audition (amongst others) in the context of mate choice and ask (3) how these neural circuits are connected to areas that have been studied in the light of multisensory integration.

## Introduction

Evolutionary fitness is not only determined by an organism’s ability to survive the world’s vagaries and dangers but also to successfully produce offspring. Finding and attracting an appropriate mating partner is of particular importance for the latter, as it determines whose genes are passed onto the next generation. Males and females have evolved different strategies to successfully select a mate, which are dependent on the expression and recognition of multiple cues belonging to diverse sensory modalities. The simultaneous diversity and species-specific nature of socio-sexual sensory cues pose a serious obstacle to the study of mate choice, possibly one of the main reasons why progress in unraveling the neural mechanisms underlying this complex behavior has been so limited.

Although communication during mate choice commonly relies on multimodal cues, there seems to be dominant, species-specific, sensory modalities. Rodents, and mice in particular, rely on pheromonal cues in order to identify their conspecifics (Luo et al., 2003), and hence a crucial role for pheromones has been postulated during mate choice in rats (Beach, 1942; Heimer and Larsson, 1967; Bermant and Taylor, 1969) and mice (Rowe and Edwards, 1972; Rowe and Smith, 1972). However, recent studies have shown that touch (Wolfe et al., 2011) and ultrasonic calls (Asaba et al., 2017) seem to be equally important during the initial social investigation and hence may be crucial for mate choice as well.

Human mate choice, on the contrary, is mostly driven by visual input (Roth et al., 2021), as gender and attractiveness can be usually identified in the split of a second. Nonetheless, other sensory modalities gain relevance as the interaction progresses, amongst which are touch and audition, similar to what has been described in rodents (Herz and Cahill, 1997; Todd et al., 2007).

In this review, we focus on the understudied impact of touch and audition in rodent and human mate choice. We begin by defining mate choice and briefly describe the main common factors that influence female and male mate choice in mice, rats, and humans. We then feature literature investigating how ultrasonic calls or voices and touch can carry relevant information during rodent and human mate choice. Finally, we elaborate on neural circuits that specifically underlie sensory processing of touch and audition in the context of rodent and human mate choice and how these neural circuits are connected to areas that have been studied in the light of multisensory integration and sexual behavior in general.

## What Is Mate Choice?

According to Halliday, (1983, p. 4) “mate choice can be operationally defined as any pattern of behavior, shown by members of one sex, that leads to their being more likely to mate with certain members of the opposite sex than with others” (Halliday, 1983). Even though same-sex mate choice is beyond the scope of this review, it is important to note that same-sex sexual behavior is omnipresent (Rosenthal, 2017, p. 11) across rodents (Coria-Avila, 2012) and humans (Gobrogge et al., 2007), hence the definition proposed by Rosenthal seems contemporarily more correct: “mate choice can be defined as any aspect of an animal’s phenotype that leads to it being more likely to engage in sexual activity with certain individuals than with others.”

Moreover, it is noteworthy to mention that mate choice is actually the outcome of two distinct components, the preference function (the order with which an individual ranks prospective mates) and choosiness (the effort to invest in mate assessment; Neelon et al., 2019). This is, an individual might not get what she/he wants if not enough effort is spent assessing conspecifics, for example. Most of the studies that we will mention in this review were performed in laboratory conditions, where very little effort is spent on mate assessment, and, therefore, we should keep in mind that some of the results and conclusions might differ in nature.

As a final note, unfortunately, many studies interrogating the influence of auditory and somatosensory cues in socio-sexual behavior are carried out in “no-choice conditions,” where a certain trait is evaluated, for example, for its influence on the detection of conspecifics, sexual motivation, and sexual arousal. Even though mate choice is not really being evaluated in these circumstances, it is reasonable to suggest that if a particular trait decreases the latency to detect a conspecific or to start having sex, it also might increase the chance that an individual will be chosen, for example, as the chooser might require less effort in assessment. Therefore, in this review we discuss some studies where the influence of a particular trait was investigated in a “no-choice condition,” keeping in mind that they are examined within this context.

Rosenthal (2017) has divided mate choice into three stages: (I) premating, which includes the detection and evaluation of all courter signals, (II) perimating, that includes behavioral patterns soon before, during, and after mating when partners are in close physical contact and (III) postmating, during which choosers can make decisions after copulation (Rosenthal, 2017, pp. 25–26). Since our review is focused on rats, mice, and humans, we will slightly deviate from this staging by substituting his term of perimating with simply mating or sex, which includes all behavioral patterns that start with the first successful mount (for rats and mice) or sexual intercourse in the case of humans. Likewise, all actions of both sexes before the first successful mount are considered as premating. The postmating stage includes all actions that are undertaken by both sexes after sperm transfer has occurred. All these three stages include the phases of: (1) receiving signals expressed by the potential mate, (2) recognizing and evaluating these signals, and (3) deciding whether to accept or reject the prospective mate.

## Female Mate Choice

The study of female mate selection has a long-standing history starting with Darwin in the 19th century (1871). His overall opinion, though, was that females succumb to their “taste for the beautiful” (Ryan, 2018) and that the male species was forced to evolve to please the female’s eye (Darwin, 1871). Fortunately, this simplistic and misogynistic view has been reformulated as a large body of literature investigating female mate choice exists not only in the context of the Darwinian sexual selection with male’s appearance and sensory cues having evolved due to female’s attractiveness (Rosenthal and Ryan, 2022) but also with a pure interest on how sensory cues (Hoier et al., 2016) and social structures (Lee and Beery, 2021) shape the behavioral pattern of female mate choice.

### Mice

Female mate choice in mice has been heavily investigated in the past decades, using well-controlled mating paradigms in the lab (Tomihara, 2005; Ganem et al., 2008; Zinck and Lima, 2013; Asaba et al., 2015; Moreira et al., 2020). These paradigms, together with initial field studies, identified a series of desired male attributes, as female mice preferred unfamiliar (Yamazaki et al., 1979; Egid and Brown, 1989; Potts et al., 1991; Penn and Potts, 1998a, b; Tregenza and Wedell, 2002; Linnenbrink and von Merten, 2017), dominant (Oakeshott, 1974; Bronson, 1979; Hurst, 1989, 1990; Mossman and Drickamer, 1996; Rich and Hurst, 1998; Rolland et al., 2003; Montero et al., 2013), healthy (Kavaliers and Colwell, 1995; Meikle et al., 1995; Zala et al., 2004; Kavaliers et al., 2006) males with significant effects for their own reproductive and sire’s fitness (Yasui, 1998, 2001; Zala et al., 2008). In accordance, female mice seem to prefer males with longer anogenital distances, a feature correlated with aggressiveness, fitness, and increased paternity (Drickamer et al., 2001). Social factors also seem to significantly impact preferences, such as the early life familial environment (Moreira et al., 2020). In the very few instances where the consequences of such mate preferences were examined, it was observed that mating with non-preferred mates had a negative impact on the progeny, as the litters from such matings had fewer pups of lower adult fitness (Drickamer et al., 2000). If possible, results obtained in laboratory settings should be confirmed in the wild, as in some cases the results can be contradictory. For example, mating with multiple males seems to be common in the wild, but rare in captivity (Hurst, 1986; Dean et al., 2006; Firman and Simmons, 2008), whose evolutionary advantages have been discussed in detail (Jennions and Petrie, 2000; Hosken and Stockley, 2003; Simmons, 2005). In contrast, mating between some closely related species only occurs in captivity (Delaney and Hoekstra, 2018). Understanding the particularities of the laboratory setting that cause such artificial preferences can however offer important insights into the underlying mechanisms. For example, inadvertent mating between closely related species was shown to be dependent on diet-based assortative mate choice (Delaney and Hoekstra, 2019), suggesting that social information is relevant, and can even override natural preferences. Hence, we encourage more field studies to confirm and/or resolve inconsistencies regarding conclusions drawn from artificial settings and as a way to explore the underlying mechanisms.

Some attempts have been made to investigate female mate choice in semi-natural environments or larger testing arenas (Hurst, 1987; Potts et al., 1991, 1994; Becker and Hurst, 2009; Montero et al., 2013; Thonhauser et al., 2013; Ruff et al., 2017) revealing female behaviors that are common in the wild, such as the so-called soliciting behavior (Bronson, 1979; Hurst, 1986). Once receptive and willing to mate, female mice express darting (the female quickly orienting towards the male and then running away) and ear-wiggling behavior (Lenschow and Lima, 2020), which seems to attract mates and increase male sexual arousal (Lee and Monks, 2013). Because of its cycles of poking and running away, female solicitation behavior leads to a paced copulation pattern whose function is yet to be investigated (Johansen et al., 2008). Old (McGill, 1962; Land and McGill, 1967; McGill et al., 1968) and recent (da Costa Araújo, 2021) studies however seem to favor the hypothesis of the existence of “a vaginal code” (Diamond, 1970), an optimal number of penile insertions performed spaced in time which might favor pregnancy. Moreover, it has been shown that female receptivity during mating is increased if the female actively initiates mating compared to when the male took the initiative, a phenomenon that is more common in larger testing arenas (Tomihara and Makino, 1991; Tomihara, 2005).

### Rats

Using comparable well-controlled laboratory settings (Lovell et al., 2007; Spiteri et al., 2012; Chu and Ågmo, 2016), a large body of literature also described female’s preference for unfamiliar (Zhang and Zhang, 2011), dominant (Taylor et al., 1982; McCormick et al., 2017; Zhang et al., 2021), and healthy male rats, whose urine contained a higher concentration of major urinary proteins (Ferreira-Nuño et al., 2005; Kumar et al., 2014). Interestingly, females seem to prefer male rats that had recently mated (Bakker et al., 1996; Galef et al., 2008), a preference that was probably mediated by odors. Newer studies, however, seem to challenge the aforementioned factors of female rat choice and deliver data suggesting that it might be random (Le Moëne and Snoeren, 2018).

Regarding choice processes during mating it has been described early on that, when possible, female rats also control or pace the rate of copulation, by performing solicitation behavior (Beach, 1976; McClintock, 1984; Erskine, 1989; Erskine et al., 1989; Pfaus et al., 2000, 2001), in the wild (McClintock and Adler, 1978; McClintock et al., 1982; McClintock, 1984) and laboratory settings (Coria-Avila et al., 2005; Guarraci and Frohardt, 2019); in fact, paced mating seems to be rewarding and can induce conditioned place preference (Paredes and Alonso, 1997), in contrast to non-paced conditions (Martínez and Paredes, 2001; Coria-Avila et al., 2005). Similar to mice, many studies also suggest the existence of a “vaginal code” (Adler, 1969; Chester and Zucker, 1970; Terkel and Sawyer, 1978; Lehmann and Erskine, 2004; Cibrian-Llanderal et al., 2010), arguing that female-paced mating has co-evolved in order to increase reproductive fitness. Female rat solicitation behavior in laboratory settings has been observed mostly during mating periods, suggesting that it enhances male sexual motivation and male arousal (Chu and Ågmo, 2016) and may signal female sexual motivation towards the male (Ellingsen and Ågmo, 2004; Sánchez Montoya et al., 2010; Santoru et al., 2014).

### Humans

Humans have adopted multiple mating strategies, and long and short-term relationships are commonly observed, even within the same individual (Schmitt, 2010). Long-term mating in humans demands extended courtship behavior, the forming of an emotional bond/love, and the investment of emotional and financial resources (Symons, 1979; Schmitt, 2010; Buss, 2013; Trivers, 2017). Numerous factors influence individual preferences in the search for long-term mating partners, such as cultural (Wellings et al., 2006; Chang et al., 2011; Kamble et al., 2014; Souza et al., 2016; Zhang et al., 2019; Walter et al., 2020), religious (Newcomb and Svehla, 1937), ethical, emotional (i.e., sense of humor, Miller, 2001), materialistic (i.e., financial resources, Daly and Wilson, 1983) factors, but also constraints (family/friends expectations) and subjective aesthetic parameters (Rusch, 2013; Huang et al., 2018). Long-term relationships, moreover, are crucial for human mate choice (both for men and women) in regard to paternity, as human offspring is born helpless, similar to rats and mice (Konig and Markl, 1987), and hence demands a time-consuming investment of parental care (Conroy-Beam and Buss, 2016). Short-term mating strategies on the contrary may rely on more immediate sensory signals, which attract and arouse the chooser (Jonason et al., 2012). Hence, factors that drive human mate choice of men and women may differ tremendously depending on whether a long or short-term relationship is sought or/and established. Long or short-term relationships might be favored at different times and also can co-exist (Buss and Schmitt, 1993; Gangestad and Simpson, 2000; Schmitt et al., 2001; Schmitt, 2005). For a detailed review of factors influencing female long-term and short-term mating strategies and their possible evolutionary implications and benefits please see Buss and Schmitt (2019).

## Male Mate Choice

Even though females have been originally thought to be choosier (Darwin, 1871; Andersson, 1994; Rosenthal, 2017), male mate choice is observed across taxa (Dewsbury, 1982; Parker, 1983; Wedell et al., 2002; Edward and Chapman, 2011; South and Lewis, 2012), including mice (Dewsbury, 1983; François Gourbal and Gabrion, 2004; Montero et al., 2013), rats (Wilson et al., 1963; Jackson and Dewsbury, 1979) and humans (Schmitt, 2010; Easton et al., 2015). Male mate choice can be based upon the specific display of female traits such as sound production in mice (Ronald et al., 2020; Sasaki et al., 2020) and the ear wiggling and hopping behavior of rats (McClintock, 1984; Martínez and Paredes, 2001) or voice pitch in humans (Moore et al., 1985; Pisanski et al., 2018). More frequent, however, is the choice of females with higher fecundity (Fitzpatrick and Servedio, 2018), which in turn can be determined by certain physical and physiological traits (Drickamer et al., 2001), such as the weight of a female mouse (Costello et al., 2009).

### Mice

Male mice are less likely to select recently mated females (Ramm and Stockley, 2014), choose non-infected partners (François Gourbal and Gabrion, 2004), that are different from their father (Beauchamp et al., 1988) and unfamiliar (Ryan and Lacy, 2003; Ramm and Stockley, 2014). Moreover, they seem to favor sequential mating under sperm competition (Dean et al., 2006; Ramm and Stockley, 2014). Interestingly, just like for females, male mate choice has been proposed to have an influence on litter size, viability, and social hierarchy (Moore et al., 2001), and the mating sequence is adapted when copulating with an unfamiliar mate (Ramm and Stockley, 2014). Regarding anogenital distance, just like females, male mice show a preference for shorter anogenital distance in females, which has been correlated with higher reproductive success and increased maternity skills (Drickamer et al., 2001).

### Rats

Very little is known regarding male mate choice in rats, even though it is well established that male rats consistently exhibit a preference for sexually receptive females (Hetta and Meyerson, 1978; Bakker et al., 1996; Bressler and Baum, 1996; Moore and Moore, 1999). Regardless of the fact that the anogenital distance in female rats seems to be correlated with lower reproductive fitness (Hotchkiss et al., 2007), this trait does not seem to affect male preference, even though the anogenital distance in male giant pouched rats is positively correlated with choosing a sexually available female (closed vs. open vagina), possibly mediated by odors (Freeman et al., 2019). Nevertheless, when comparing experiments in the lab with semi-natural conditions, copulatory patterns and preferences may change. While in standard small test cages male rats achieve 7–9 ejaculations before reaching sexual exhaustion (Beach and Jordan, 1956; Tiefer, 1969; Rodríguez-Manzo and Fernández-Guasti, 1994), much fewer (3–4) ejaculations are reported when tested in semi-natural conditions (Chu and Ågmo, 2014; 2015a,b). Moreover, male rats barely copulate with females that are not fully receptive in a semi-natural environment (Chu and Ågmo, 2015a,b), but do so in a small testing arena (Madlafousek and Hlinak, 1977; Spiteri and Ågmo, 2006; Oliveira et al., 2021).

### Humans

Similar to women, male mate choice can vary depending on the desire to establish a short or long-term relationship (for an in-depth opinion on men long-term and short-term mating please refer to Easton et al., 2015; Buss and Schmitt, 2019).

## Contribution of Ultrasonic Vocalizations to The Evaluation and Choice of Mating Partners (Premating)

### Mice

As for any other behavior, animals can gather information from all sensory modalities in order to choose a potential mate. Without doubt (Beach, 1942), olfactory information is crucial for rodent mate choice (Coombes et al., 2018; Ferkin, 2018) and odors carry important information about sex (Johnston, 2003; Choleris et al., 2009; Hurst, 2009; Kondo and Hayashi, 2021), strain (Krackow and Matuschak, 1991; Laukaitis et al., 1997; Bímová et al., 2009; Zinck and Lima, 2013), social rank (Jemiolo et al., 1985, 1991; Zhang et al., 2021), sexual receptivity (Novotny et al., 1990; Dulac and Torello, 2003), fitness (DeFries and McClearn, 1970; Oakeshott, 1974; Gill and Rissman, 1997; Temple et al., 2002), and health status (Kavaliers and Colwell, 1993; Zala et al., 2004; Kavaliers and Choleris, 2017) of conspecifics. Indeed, several studies describe a prominent role for pheromones during the initial sexual approach in rats (Carr, 1974; Kumar et al., 2014; Zhang et al., 2021) and mice (Roberts et al., 2010, 2014; Haga-Yamanaka et al., 2014; Demir et al., 2020). For a detailed recent review about the impact of pheromonal information and the underlying neural circuitry during mate choice in rodents, please refer to Stowers and Liberles (2016) and Deangelis and Hofmann (2020).

**Figure 1:**
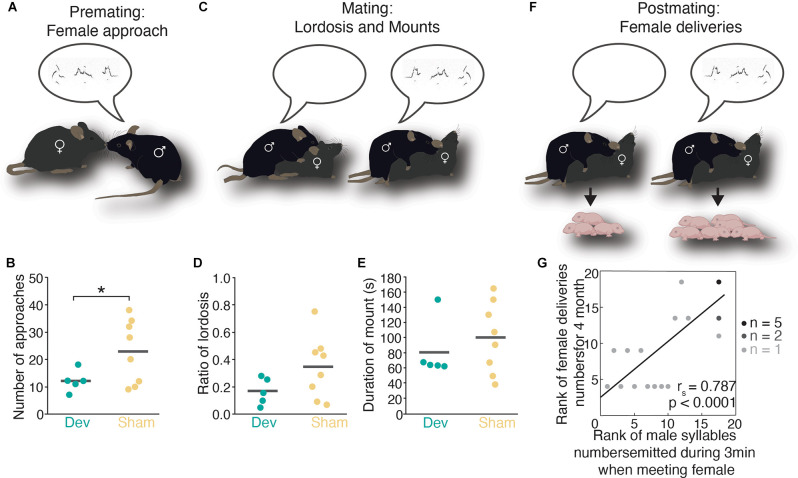
Male mouse ultrasonic vocalizations and its impact on female mate choice during premating, mating, and postmating. **(A)** Male USV calls emitted during premating elicit female approach behavior. **(B)** Number of female approaches is significantly higher when the male sings compared to devocalized males. **(C)** Male USVs emission during mating leads to an increase in lordosis behavior. **(D)** Ratio of lordosis is higher in females than received mounts and intromission from males that emitted USVs compared to males that were devocalized. **(E)** The duration of mounts in devocalized or intact males is not different. **(F)** Female mice deliver more pups when mated with males that emitted USVs. **(G)** A significant correlation between the number of male USVs and female deliveries has been found. Panels **(B,D,E,G)** adapted and reprinted with permission from Asaba et al. (2017). *indicates significant differences (*p* < 0.05) betweendevocalized male and sham-operated (Student’s t-test).

Pheromones may be the most important modality for the initial approach, but they are most certainly not acting in isolation (Asaba et al., 2014, 2017; Ågmo and Snoeren, 2017; Haskal de la Zerda et al., 2020; Ronald et al., 2020; Zala et al., 2020; Contestabile et al., 2021). However, it has been hard to unravel the role of other cues in mate choice; controversy surrounds the role of calls for example, in part due to the difficulty of identifying who says what, an obstacle that was overcome by recent technical advances (Binder et al., 2020) in the capacity to locate sound sources (Neunuebel et al., 2015; Heckman et al., 2017; Warren et al., 2018; Coffey et al., 2019; Sangiamo et al., 2020; Oliveira-Stahl et al., 2021). Hence, recent studies in mice (Asaba et al., 2017; Ronald et al., 2020; Warren et al., 2020) made important progress towards investigating the role of sound communication during various socio-sexual behaviors (Sangiamo et al., 2020) and found that specific calls in the ultrasound range (named ultrasonic vocalizations, USVs) are expressed by males and females during mating (Holy and Guo, 2005; Potfors and Perkel, 2014; Heckman et al., 2016; Seagraves et al., 2016; Asaba et al., 2017; Matsumoto and Okanoya, 2018; Niemczura et al., 2020; Ronald et al., 2020; Warren et al., 2020). Wild (Musolf et al., 2010) and laboratory (Chabout et al., 2015, 2017) male mice produce a courtship song, specific to their genetic background (Sugimoto et al., 2011; Melotti et al., 2021) and adapt their USVs to the reproductive state of the female (Hanson and Hurley, 2012) indicating that odors may affect USVs production by directing courtship behavior to females that are likely to mate. Interestingly, female mice also express a complex courtship song that may signal her hormonal receptivity (Neunuebel et al., 2015) when animals initially explore and chase each other (Lenschow and Lima, 2020). Female mice show a preference for calling males (Pomerantz et al., 1983; Nomoto et al., 2018; Tschida et al., 2019), especially those that emit high complex calls over simpler ones (Chabout et al., 2015; Matsumoto and Okanoya, 2018), and male USVs enhance female approach behavior (Hammerschmidt et al., 2009; Asaba et al., 2017; Figures 1A,B). Male and female seem to increase the rate of call production when they are further away from each other, indicating that both sexes use USV to attract mates (Warren et al., 2020). Moreover, they postulate that “it may be evolutionarily advantageous for male mice to alter their vocal emissions to (travel/overcome) greater distances, as this adaptation may enhance the likelihood to sire offspring”. Regarding female approach behavior, a recent study showed that males produce complex songs in response to female urine, further hinting that pheromonal cues may act as triggers for USV production, intensifying female attraction towards the male (Ronald et al., 2020). Interestingly, female USVs are not exclusively triggered by male urine (Maggio and Whitney, 1985), suggesting that in the wild it might be the male that is chosen by the female once he starts calling due to the encounter with female urine (Nunez et al., 1985).

While USVs may serve to advertise sexual receptivity (Neunuebel et al., 2015), the interactive nature of these cues was demonstrated by the observation that male and female mice simultaneously decrease the rate of USV production during chasing behavior and that males increase the production rate when females run away (Finton et al., 2017; Warren et al., 2020). Warren et al. (2020) complement this study by showing that male mice clearly adapt their courting behavior to the calling female by accelerating when the female calls from further away. Similar to other cues, it has been shown that USVs can be used for inbreeding avoidance, in BL6 (Musolf et al., 2010, 2015; Asaba et al., 2014; Nomoto et al., 2020) and wild type mice (Hoffmann et al., 2012; Nicolakis et al., 2020), thereby impacting the progeny’s fitness.

Likewise, male USVs contain information about their social rank (Nyby et al., 1976; D’amato, 1991; Pasch et al., 2011) as dominant males exhibit higher frequency calls in the presence of a female compared to lower rank individuals, most likely *via* a testosterone dependent mechanism (Nunez et al., 1978; Kikusui et al., 2021).

The impact of pheromonal cues on female sexual receptivity has been described early on, with the identification of pheromones capable of triggering endocrine responses in females, i.e., increased sexual receptivity (Whitten effect; Whitten, 1958), earlier onset of puberty (Vandenbergh, 1969) and pregnancy block (Bruce effect; Bruce, 1959). Whether male mice USVs influence female receptivity, as described for songbirds (Leboucher et al., 1998; Bentley et al., 2000), has been scarcely examined, but a recent study indicates that male calls can indeed trigger female fertility, most likely through the activation of central kisspeptin neurons (Asaba et al., 2017).

Female mice also emit audible cues, known as squeaks (Wang et al., 2008), when interacting with males. Squeaks have been shown to be emitted during rejection behavior (Sugimoto et al., 2011; Finton et al., 2017), but also when a female accepts the male’s attempt at copulation (White et al., 1998; Finton et al., 2017), complicating efforts to understand their meaning. Male USVs, however, are dependent on the particular context the female squeak is emitted: while they stop calling when females squeak during rejection behaviors, they increase their courtship song when the female squeaks and allows them to mount (Grimsley et al., 2013).

### Rats

USVs in rats have been classified into pleasure or reward calls that occur in the 50 kHz range (Wöhr, 2018; Berz et al., 2022) and alarm or fear calls that are emitted in the 20 kHz range (Lenell et al., 2021). Similar to mice, rat calls contain information about dominance status (Xiao et al., 2004; Portfors, 2007) and genetic background (Sales, 1979), results that have been recently confirmed (Bogacki-Rychlik et al., 2021; Berz et al., 2022). Interestingly vocalizations may signal sickness in rats as well (Kirsten et al., 2015).

Like in mice, female rats emit more 50 kHz calls when sexually receptive (Thomas and Barfield, 1985; Matochik and Barfield, 1994; Bernanke et al., 2021; Lenell and Johnson, 2021) and males increase their calling rate in the presence of a receptive female (McGinnis and Vakulenko, 2003; Portfors, 2007) indicating the potential of USVs to modulate female mate choice. However, there has been controversy regarding the importance of male calls in female mate choice; while some studies support the importance of male calls (Thomas and Barfield, 1985; Gerson et al., 2019) eliciting female approach behavior (Seffer et al., 2014; Willadsen et al., 2014; Berg et al., 2018, 2021; Kisko et al., 2018, 2020), others found that females choose vocalizing vs. non-vocalizing males equally often (Snoeren et al., 2014). Despite the contradictory results, it is well accepted that female rats emit more calls when interacting with male vs. female conspecifics (White et al., 1993; Armas et al., 2021), probably signaling sexual motivation towards the male (Börner et al., 2016) and that male USVs can trigger female solicitation behavior (McIntosh et al., 1978), including ear wiggling and darting behavior. Solicitation behaviors seem to be rewarding for both sexes (Martínez and Paredes, 2001) and increase male sexual arousal, hence activating male mounting behavior with a shorter latency (Chu and Ågmo, 2015b; Ågmo and Laan, 2022) and pointing to a certain choice from the male towards the female.

Unlike female mice, female rats increase their calling rates when presented with male odors (White et al., 1993), but no impact of these calls on approach behavior (Seffer et al., 2014; Willuhn et al., 2014; Brenes et al., 2016) or other copulatory parameters has been observed (Snoeren and Ågmo, 2013; Snoeren et al., 2014; Ågmo and Snoeren, 2015) questioning the role of female calls. Nevertheless, male rats increase the rate of USVs when they expect to interact and copulate with a female, and their number of calls was positively correlated with the number of ejaculation (Bogacki-Rychlik et al., 2021) supporting a communicative value of male calls during premating and mating contexts. Another recent study showed that rats of both sexes are attracted by 50 Hz calls in general and will approach the sound source (Davidson and Hurst, 2019). However, it must be noted that most rat sexual behavioral studies were performed in a laboratory setting and the social structure differs substantially from what is observed in natural conditions (McClintock, 1984). Wild rats usually live in a complex burrow system with multiple female and male rats, forming groups ranging from 7 to 100 individuals and most likely sexual behavior is different (Steiniger, 1950; Calhoun, 1962; Telle, 1966). Some researchers favor mate choice in the wild (McClintock and Adler, 1978), while others do not (Schweinfurth, 2020).

Automated behavioral tracking systems and state-of-the-art sound localization tools to monitor rat sexual behavior in a large group of individuals in the natural environment (Ventura-Aquino and Paredes, 2020) are needed in order to disentangle whether USVs serve as a crucial modality during the initial phases of mate choice.

## Contribution of Ultrasonic Vocalizations During Mating and Postmating Choice

### Mice

Besides an impact on precopulatory mate choice, USVs also seem to affect on-going copulatory mate choice as females express a less drastic lordosis posture when mating with a silent male (Asaba et al., 2017, Figures 1C–E). One of the most striking results regarding the role of USVs during mating originates from the same study where it was reported that female mice who have copulated with males that constantly sang their harmonic songs during sex (Matsumoto and Okanoya, 2016) produced more numerous litters than females that mated with males that emitted less or no calls during copulation (Asaba et al., 2017, Figures 1F,G). This phenomenon definitely deserves more attention as it would be interesting to test if a female aborts ongoing copulation with a silent male when other options are available, for example. If a similar effect exists in rats is to date unknown.

Also, during post-copulatory mate choice, USVs could act in favor of mate choice since there is evidence for a more frequent emission of a 40 kHz call in the last mount immediately prior to ejaculation (pre-ejacultory call; White et al., 1998) while USV production seems to cease immediately after ejaculation (Sales and Pye, 1974; Nyby, 1983; Wang et al., 2008) in mice.

### Rats

Male rats (Barfield and Geyer, 1972) emit high-frequency 20 kHz calls (Burgdorf et al., 2008) once they enter the phase of sexual satiety. These 20 kHz calls have been classically described as fear calls since they most certainly signal alarm or danger to conspecifics (Wöhr, 2018). Why they are also emitted by a sexually sated male rat remains unclear, but the most obvious reason might be that the 20 kHz calls reflect a withdrawn state that is similar to a dangerous/fearful or sexual satiated context (Anisko et al., 1978; Adler and Anisko, 1979). Nevertheless, it has been proposed that they could also serve to maintain female contact and probably discourage other males to copulate with the “chosen” female (Barfield and Geyer, 1972, 1975; Anisko et al., 1978; Adler and Anisko, 1979; Thomas et al., 1982; Wöhr, 2018). Another hypothesis suggests that these calls may transmit dominance information, which could be used by the female to orient her willingness to mate towards more dominant males vs. subordinate, as it has been shown that females who have received an ejaculation from a subordinate male resume mating quicker when compared to an ejaculation from a dominant one (McClintock et al., 1982). It remains to be investigated whether the post-ejaculatory song differs between subordinate vs. dominant males, although early studies suggest that these calls when originating from subordinate males are longer (McClintock et al., 1982).

Similar to mice, a pre-ejaculatory call also seems to exist in male rats (White and Barfield, 1990). Whether these pre-ejaculatory calls are crucial either to signal mate-choice to surrounding male competitors or serve as a signal to inform the female of an approaching ejaculation remains to be determined. Still, considering that female rats and mice need a minimum amount of vaginal stimulation in order to trigger the necessary neuroendocrine mechanisms for pregnancy establishment (Adler, 1969; Diamond, 1970), we can hypothesize that these pre-ejaculatory calls may be used by the female to abort the approaching ejaculation in situations where she did not receive sufficient vaginal stimulation. Supporting this idea, it has been shown that female rats usually increase their calling rates when male mating calls are presented *via* playback, but do not do so when pre-ejaculatory calls are played back (White et al., 1993).

Finally, recent studies in rats and mice have shown that USVs of the opposite sex increase the sexual motivation of both male and female rats (Bialy et al., 2019; Lenell et al., 2021) and mice (Pomerantz et al., 1983; Finton et al., 2017; Fernández-Vargas, 2018; Kuwaki and Kanno, 2021; Zhao et al., 2021). Sexual motivation in turn has been shown to be a strong reinforcer for mate choice (Matthews et al., 2005; Bialy et al., 2019).

## The Impact of Somatosensation for Rat and Mouse Mate Choice (Premating)

Species with low visual acuity, including rats and mice (Jennings et al., 1998; Minini and Jeffery, 2006) are often nocturnal (Barnett and Bathard, 1953; Ahl, 1986; Schweinfurth, 2020), relying on smell and other modalities, such as touch (Ahl, 1986; Lenschow et al., 2016) to navigate the world. Large facial whiskers, capable of moving in specialized and complex ways (referred to as whisking), gather most of the touch information needed to parse textures and avoid obstacles (Brecht et al., 1997), and evaluate conspecifics (Barnett and Bathard, 1953; Wolfe et al., 2011; Bobrov et al., 2014; Lenschow and Brecht, 2015). Other smaller, touch-sensitive body hairs transmit information when moving along walls or other obstacles (Latham and Mason, 2004), and might be substantially activated when animals groom each other. Touch is omnipresent when rodents socially interact in close proximity, playing a role in all sorts of behaviors, from aggression to parental care and play behavior (Barnett and Bathard, 1953). Maybe not that surprising, social and non-social touch are accompanied by distinct whisker movements and neuronal responses (Bobrov et al., 2014; Rao et al., 2014; Lenschow and Brecht, 2015), with social touch leading to small-amplitude, irregular patterns that evoke much stronger responses, when compared with other non-social objects (Bobrov et al., 2014). Thus, even though understudied, it seems reasonable that social touching, independently of which body part is scanned, may carry important information during sexual behavior and mate choice. In fact, several observations support a role for whisker touch and its integration with other sensory modalities in the context of mate choice in rodents, as anogenital investigation, a key source of smells for mate assessment, is oftentimes accompanied by intense whisker movements (Wolfe et al., 2011).

### Mice

Whisking during social interactions in male mice is scarcely studied, even though the so-called barbering behavior is known for a long time: male and female mice trim the whiskers of their cage mates, a sign of dominance (Long, 1972) and hence could also signal a certain fitness during mate selection. Interestingly, whisker trimming was also observed among mated pairs with the male trimming the female until she became pregnant (Long, 1972). To what extent this whisker trimming could indeed signal fitness or even be a post-copulatory mate choice mechanism (“this female is mine”) deserves further attention in multiple partner-choice paradigms. Interesting questions arise from all these studies and it would be particularly crucial to investigate whether whisker-clipped males or females are less attractive.

### Rats

Social whisking in Long Evans rats exhibits several instances of sexual dimorphism, with male rats holding their whiskers more protracted but whisking similarly when interacting with both sexes, while females whisk with smaller amplitudes when interacting with males compared to females (Wolfe et al., 2011). In this same study (Wolfe et al., 2011) the exact position of the vibrissae was shown to signal aggressiveness. An earlier study with rats that employed burrows, to mimic natural conditions, reported that some individuals retract their whiskers immediately after having made contact with the facial hairs of a defender and that females may use that signal to protect other non-receptive females from being mounted by males (Blanchard et al., 2001). Hence we hypothesize that females or even males may transmit their non-readiness to mate *via* a specific position of their vibrissae. To what extent these tactile cues are indeed actively involved in mate choice needs further research in a more naturalistic setting.

A potential function of vibrissae in the dispersion of pheromones has been suggested early on (Marler and Hamilton, 1966; Ahl, 1986) but never investigated in fine detail. The fact that whisking and sniffing are strongly correlated and operate at the same frequency (4–12 Hz; Welker, 1964; Ranade et al., 2013) supports a synergistic action of touch and smell together with audition, as USVs have been shown to be emitted during active sniffing periods (Roberts, 1972; Riede et al., 2011; Sirotin et al., 2014), locked to exhalation (Riede, 2014; Boulanger-Bertolus and Mouly, 2021). As already mentioned, a possible synergistic interaction between touch and smell becomes even more apparent after the initial approach takes place: male and female mice, and rats, not only closely investigate each other’s faces but also sniff and whisk all over their bodies and most importantly their anogenital region (Lenschow and Lima, 2020). To what extent facial hairs are needed to transmit or amplify the odor information is not known.

## Contribution of Somatosensation During Mating and Postmating Choice

Genital tactile stimulation (Paredes and Alonso, 1997; Paredes and Vazquez, 1999; Meerts and Clark, 2009; Parada et al., 2010, 2011) and paced mating (Guterl et al., 2015) are able to trigger conditioned place preferences (CPP) in female rats (reviewed in Pfaus et al., 2016) and, therefore, might be an important cue during mate choice, especially during the phases of on-going and post-copulatory mate choice. Indeed, artificial and experimentally controlled vaginocervical (Meerts and Clark, 2009) and clitoral stimulation are able to induce CPP (Cibrian-Llanderal et al., 2010; Parada et al., 2010) which was blocked by pharmacological (Meerts et al., 2015) or nerve ablation experiments (Clark et al., 2011). Interestingly they also suggest that only clitoral stimulation is crucial for the development of paced mating (Meerts et al., 2010, 2015; Parada et al., 2010). In addition, clitoral stimulation was shown to trigger 50 kHz calls in hormonally primed female rats (Gerson et al., 2019) indicating the positive valence of this type of genital touch. In male rats, vaginal thrusting, and presumably penile stimulation alone, are able to trigger conditioned place preference but only if they have not experienced ejaculation before (Tenk et al., 2009).

Mice studies investigating the valence of genital stimulation are so far absent.

The female’s solicitation behavior is activated by male tactile stimulation (chasing, touching the flanks, and anogenital sniffing) and this has been particularly well described for rats (Ågmo et al., 2004; Ågmo, 2008; Chu and Ågmo, 2016). To what extent a display of solicitation behavior has an impact on ongoing copulatory mate choice or even its impact on reproductive fitness is not known so far.

Genital stimulation during copulation is an interactive and rewarding process that ensures that sperm is transferred, and pregnancy initiated. As previously mentioned, spaced sensory vaginal stimulation, seems to be crucial for pregnancy success and outcome in rats and mice (de Catanzaro, 1991; Ventura-Aquino et al., 2018), suggesting that genital sensory stimulation could be a potential mechanism for female mating and postmating choice (Brennan and Prum, 2015). It would be particularly interesting to investigate whether female rats resume mating quicker if they have not received sufficient vaginal stimulation or whether they prefer long-ejaculating males over fast-ejaculators (Pattij et al., 2005).

Male mice and rat genitalia consist of a baculum (penis bone) whose morphology is associated with higher male fitness (Ramm, 2007; Ramm et al., 2010; Stockley et al., 2013; Simmons and Firman, 2014; André et al., 2018; Winkler et al., 2021) and males underlying higher sexual selection pressure have a thicker baculum (Stockley, 2012; Stockley et al., 2013). A thickened baculum enabling greater mechanical stimulation of the female tract could serve an increase in female fertilization in two ways (Simmons and Firman, 2014): first, it may promote greater neuroendocrine responses in order to prepare embryo implantation and subsequent offspring development (Eberhard, 1996; Stockley et al., 2013); second, it could stimulate greater oviductal secretion enhancing sperm rheotaxis (Miki and Clapham, 2013) towards the uterus. Interestingly, male mouse genital stimulation, on the other hand, seems to contribute differently to reaching the ejaculatory threshold, as when mating with a non-preferred female mouse the copulatory sequences are altered, with males performing more mounts with intromissions (Ramm and Stockley, 2014) indicating that more sensory stimulation is needed in order to reach ejaculation.

The baculum serves as well to form the copulatory plug in rats and mice (Winkler et al., 2021), a thick mass that the male deposits at the end of ejaculation, which basically prevents remating of the female with other male competitors (Voss, 1979; Ramm et al., 2005; Schneider et al., 2016; Sutter and Lindholm, 2016; Sutter et al., 2016) or in situations when the female remates, it prevents sperm transfer of the competitor (Stockley et al., 2020). The capacity to remove the plug is thought to depend on the baculum morphology as well (Simmons and Firman, 2014; André et al., 2018; Winkler et al., 2021).

## Synergistic Action of Multisensory Cues in Mate Choice of Mice and Rats

Multi-sensory dependent perception and communication is ubiquitous during socio-sexual encounters and may direct the initiation, maintenance and finalization of any particular behavior in many different ways (Chen and Hong, 2018). In fact, mate choice during all three stages may be based on decoding of multimodal cues whereas it is often unclear to what extent these cues carry non-redundant or redundant meanings, thereby either complimenting a decision to mate or compete for a given outcome (Johnstone, 1995; Johnstone et al., 1996; Ronald et al., 2012; Halfwerk et al., 2019). While the large body of literature investigating the role of single sensory modalities during mate selection delivered important insights into which sense might be the main driver for different mate choice phases, i.e., initial approach, it has blunted our understanding of how differential processing contributes to female and male mate choice.

### Rats

Stone (1922) and Beach (1942) postulated early on that more than one modality is necessary to trigger male sexual arousal and approach behavior thereby resulting in copulation (Stone, 1922; Beach, 1942). These early studies have been complemented by Ågmo and Snoeren (2017) who suggest that the initial approach behavior of a male rat towards a female is induced by the combination of at least two sensory modalities (olfaction, vision, and others with the latter testing an anosmic male in the dark) whereby olfaction seemed to be crucial. They concluded the existence of a cooperative function and a resultant summation of sensory modalities. While the latter rat study supports the notion that USVs indeed seem to play a minor role during rat approach behavior (Heinla et al., 2021), it cannot be ruled out that they may carry incentive or decisive value once two rats facially touch each other (Rao et al., 2014), giving rise to the possibility of either redundant or non-redundant multi-modal signaling.

### Mice

Female USVs seem to be crucial for male mice approach behavior, prompting males to start singing their courtship song. Interestingly the latter only occurs when males are presented with female USVs and urine together suggesting an underlying multi-sensory integration process giving rise to non-redundant signals that lead to distinct behavioral outcomes (Ronald et al., 2020). Likewise, it was shown that male mice USVs and urine act synergistically to attract female mice (Wang et al., 2008; Asaba et al., 2014, 2017) and that close tactile contact is needed for male mice to modulate their USV song towards the female (Wang et al., 2008) and that they continue vocalizing to female urine when a realistic female encounter was given a forehead (Zala et al., 2020).

The process of familial and sexual recognition is in particular essential for premating choice processes and a comparative study between mice and rats shows that while olfaction indeed seems to be the main sensory modality needed for recognizing the opposite sex, the ability to recognize a familiar individual is greatly impaired when clipping the whiskers or impairing the hearing sense (Haskal de la Zerda et al., 2020). Even though it was not studied if the male rats and mice would have equally copulated with an unfamiliar vs. a familiar conspecific, it shows that social recognition by rats and mice relies on the integration of several sensory modalities. In line with this study is the finding that multiple sensory modalities indeed seem to represent a more salient stimulus in comparison to when only an odor stimulus is presented (Contestabile et al., 2021).

All aforementioned studies exclusively investigated multi-modal processing during male towards female approach, which is somewhat surprising given the fact that the longstanding notion is that females are the choosing sex (Rosenthal, 2017).

## The Importance of Audition and Somatosensation During Human Mate Selection

### Audition during pre-mate choice

**Figure 2:**
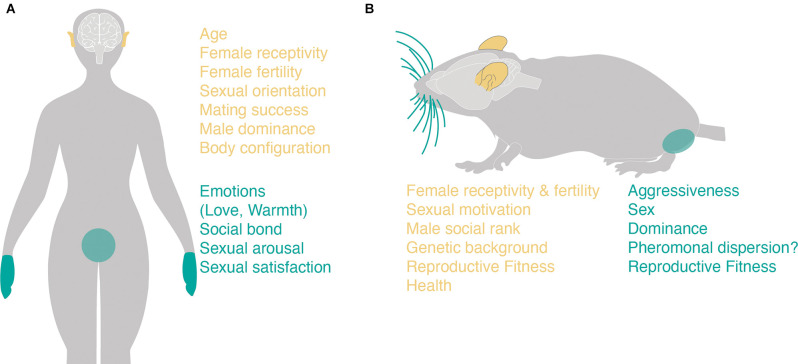
Potential roles of touch and audition during human and mice/rat mate choice. **(A)** The parameters that are transmitted *via* differential voice features in humans (left panel, human ears) are written in yellow (right panel) whereas those that can transmit differential valence of socio-sexual touch (right panel, hands, and genitals) are highlighted in turquoise. **(B)** Same as **(A)** but for mice (upper panel). The different social cues or internal states that can be transmitted *via* ultrasonic vocalizations in mice are highlighted in yellow (lower left panel) whereas those that may be communicated *via* socio-sexual are written in turquoise (lower right panel).

The sense of vision can be seen as the human counterpart to the rodent pheromone’s decisive cue during mate choice. In fact, visual attractiveness has been widely studied for human sexual selection and this in particular is the case for short-term mating (Buss and Schmitt, 2019). Tactile and auditory modalities have been largely neglected, even though the latter is a sex-defining sensory cue (Puts et al., 2006, 2012). The human voice transmits various and differential social cues just as for mice and rats (Figure 2) and hence age (Ptacek and Sander, 1966; Linville and Fisher, 1985), sexual orientation (Munson et al., 2006), sexual receptivity (Bryant and Haselton, 2009; Fischer et al., 2011; Klatt et al., 2020), and fertility in women (Feinberg et al., 2006; Pisanski et al., 2018), dominance in men (Puts et al., 2007, 2012; Cheng et al., 2016; Schild et al., 2020), physical strength in men (Sell et al., 2010; Schild et al., 2020), and body configuration in men and women (Hughes et al., 2004; Rendall et al., 2007; Pisanski and Rendall, 2011) can all be decoded from human voice. Since human mate choice can be based on all of these signals, the voice might be, just like vision, a crucial modality to choose a potential mate.

The voice is most certainly a sex-defining sensory modality as a broad body of literature exists reporting that men speak at a lower vocal pitch than women (Fitch and Holbrook, 1970; Childers, 1991; Puts et al., 2012; Titze, 2017). Most men prefer women with higher pitch voices (Collins and Missing, 2003; Feinberg et al., 2008; Jones et al., 2008; Apicella and Feinberg, 2009; Puts et al., 2011; Abend et al., 2015) particularly for short-term mating (Puts et al., 2011) while women prefer men with lower pitch voice (Collins, 2000; Feinberg et al., 2005, 2006, 2008; Puts et al., 2006, 2007; Jones et al., 2010), although this might also only occur when looking for short-term partners (Puts, 2005; Jones et al., 2010). Furthermore, certain voice features, such as volume or speech duration, are correlated with a higher number of sexual encounters and mating success for both men and women (Hughes et al., 2004; Puts, 2005; Puts et al., 2006; Apicella et al., 2007; Hodges-Simeon et al., 2011; Atkinson et al., 2012; Suire et al., 2018). Impressively, the women’s voice pitch is also modulated by their menstrual cycle (Bryant and Haselton, 2009; Fischer et al., 2011; Klatt et al., 2020) and the latter impacted their preference for men’s voices (Puts, 2005). Klatt et al. (2020) recorded naturally cycling women, during the high and low fertility phases of the cycle, saying neutral content or sentences associated with social events, namely mate choice. After hearing the recordings, men showed a clear preference for the social content recordings, especially if this came from women in the high fertility phase, who also had a slightly higher pitch (Klatt et al., 2020) confirming findings of an earlier study (Pipitone and Gallup, 2008). During menstruation on the contrary men find women’s voices least attractive (Nathan Pipitone and Gallup, 2012).

Most of these studies were based on questionnaires after having presented recorded files. Pisanski et al. (2018) described voice modulation changes in a natural setting in which subjects underwent real speed-dating events and found similar results: men lowered their pitch when interacting with highly desirable women, especially if they considered the women as a potential mate and the choice was reciprocated. Interestingly, women had the opposite behavior: when in the presence of their chosen potential mates, women showed a higher and more variable pitch (Pisanski et al., 2018).

### Audition during mating and post-mating choice

Whether men’s and women’s voices during mating, especially orgasm, and post-mating are different and whether these modulations can impact reproductive fitness comparable to rodents is not known.

### Somatosensation during pre-mate choice

As for mice and rats, touch can have differential valence for humans (Figure 2). Discriminative touch is used to detect shapes and textures of objects or surrounding nature, whereas affective touch or social touch is crucial to assess interactions with other humans. Social touch perception can range from “orgasmically pleasant to excruciatingly unpleasant” (Cascio et al., 2019) depending on context (Saarinen et al., 2021), culture (Hall, 1996; Field, 2010), sexuality, and gender (Morrison et al., 2010; Ellingsen et al., 2016). Early studies strongly suggest that affective touch can transmit many emotions (Hertenstein et al., 2006), amongst which are friendship and warmth (Mehrabian and Epstein, 1972) but also sexuality and intimacy (Jourard and Rubin, 1968; Heslin, 1974; Heslin et al., 1983). In fact, in intimate relationships, social touch plays a crucial role, and simple actions, like holding hands, can result in a sensation of relief during painful situations (Goldstein et al., 2018). As humans reach sexual maturity, social touch gains a sexual and romantic valence, and becomes relevant in the process of choosing a partner (Major et al., 1990) such as hand-holding during flirting (Moore, 1985; Eibl-Eibesfeld, 1989; Lee and Guerrero, 2001). Even though men seem to value tactile cues more than women in the context of premate choice (Herz and Cahill, 1997) another study on men and women showed that various emotions, including love, can be detected by being touched by an unknown person (Hertenstein et al., 2009) and affective touch is important for both genders to form stable and secure relationships (Suvilehto et al., 2015; Krahé et al., 2018). Early studies hint that women were more likely to receive touch from men while men tended to initiate touch towards the opposite sex (Henley, 1973; Stier and Hall, 1984; Moore, 1985; Major et al., 1990; Hall, 1996). Women were more prone to same-sex touch (Stier and Hall, 1984) and not proactive in touching men; a finding that may be explained with men overinterpreting women signals in a mating context, and this has been in particular clear for touch (Struckman-Johnson and Struckman-Johnson, 1993). These sex differences in affective touch have been confirmed by recent studies (Schirmer et al., 2021, 2022). Moreover, it is noteworthy that the touch of different body parts signals various context and relationship-dependent meanings (Jones and Yarbrough, 1985; Routasalo and Isola, 1996). Whereas touching the arms and hands is considered mostly neutral, touching other parts of the body can mean and be perceived in several different ways, that are context-dependent and can range from positive (Willis and Briggs, 1992; Suvilehto et al., 2015) to highly unpleasant and intrusive (Lee and Guerrero, 2001). While a causal role of social touch for short-term partner choice might be questionable (Herz and Cahill, 1997), it gets clear that the quality of affective touch is tightly linked to relationship satisfaction in adult romantic couples (Gulledge et al., 2003; Hertenstein et al., 2006; Wagner et al., 2020) and, therefore, might be an important factor for long-term mate choice.

### Somatosensation during mating and postmating

Somatosensation is evaluated during or after sexual intercourse (mating and postmating) and is a great modulator of sexual arousal (Georgiadis et al., 2012). Pleasurable penile stimulation during intercourse most likely favors female orgasm (Schultz et al., 1999), which in turn has been proposed to be important to produce an “upsuck” response, thereby transporting sperm through the cervix into the uterus (Fox et al., 1970; Baker and Bellis, 1993a, b; Baker et al., 1996; Faix et al., 2001; Meston et al., 2004). Hence, penile morphology leading to adapted sensory stimulation of the vagina and clitoris during intercourse might be a criterion for post-copulatory mate choice in women and by rendering it rewarding or satisfying, reproduction may be enforced. In fact, women value the thickness and length of a partner’s penis as significant impact in their sexual satisfaction (Štulhofer, 2006). Despite penile sensory stimulation, it must be mentioned, however, that other objects or practices of clitoral stimulation during human sex may affect arousal and orgasm outcome (Pfaus et al., 2016).

## Synergistic Action of Multisensory Cues in Human Mate Choice

One of the first descriptions of different valences associated with various sensory modalities during human mate preferences was reported by Herz and Cahill (1997), who developed a questionnaire entitled “Sensory Stimuli and Sexuality Survey”. The questions were focused on the subjects’ preference for auditory, visual, olfactory, and tactile stimuli when choosing a lover, engaging in sexual activities, or in a neutral task. During the initial evaluation of attractiveness, men valued smell and visual stimuli equally, whereas women had a strong preference for olfactory cues over any other stimuli. Moreover, women showed no preference for touch or the partner’s voice, while men rated touch over voice. However, when asked for their preference during sexual intercourse, men still preferred visual stimuli, but gave equal importance to touch, followed by sexual sounds, with olfaction last in the line of preferences. Females, on the other hand, rated touch during sex as the most preferred stimuli, followed by visual, sexual sounds, and olfaction (Herz and Cahill, 1997). Sorokowska et al. (2018) examined the differences in preferred sensory modality between blind and sighted subjects. Interestingly, blind people showed a strong preference for audition, whereas, in the sighted group, smell was the most valued, similarly to Herz and Cahill (1997). Even more striking was that all subjects, but blind subjects specifically, found touch significantly less meaningful when compared to smell or audition. Furthemore, women tended to give more importance to smell and audition, whilst men rated smell over all other modalities (except for blind men that valued audition more; Sorokowska et al., 2018). Another study asking blind subjects to rate sensory modalities, found contradicting results with voices being the most preferred modality (Scheller et al., 2021) in men and women. Nevertheless, all these studies show that there is an integration of various sensory modalities when performing mate choice and that when one modality is unexpectedly eliminated, others will become more salient.

Moreover, it seems that vision and audition are particularly redundantly integrated, as high pitch voices of women were correlated with how visually attractive men also considered the women (Collins and Missing, 2003; Jones et al., 2008; Abend et al., 2015).

We must stress that there are very few studies examining the multimodal effect of human mate choice and those that exist did not manipulate the weight of one modality vs. the other. More research should be inspired by a recent study by Roth et al. (2021) during which they examined the predictive power of vision, scent, and voice-sound to lead to further dating in a natural setting (Roth et al., 2021). It would be interesting to see if their finding of vision being the strongest predictor of attractiveness (auditory and olfactory showed small to no effects) can change when participants were treated with an unpleasant smell or if the pitch of their voices were artificially manipulated.

## Neural Circuits for Auditory and Somatosensation in The Context of Mouse and Rat Mate Choice

### Neural circuits for USV detection and production and their implications for mate choice

**Figure 3:**
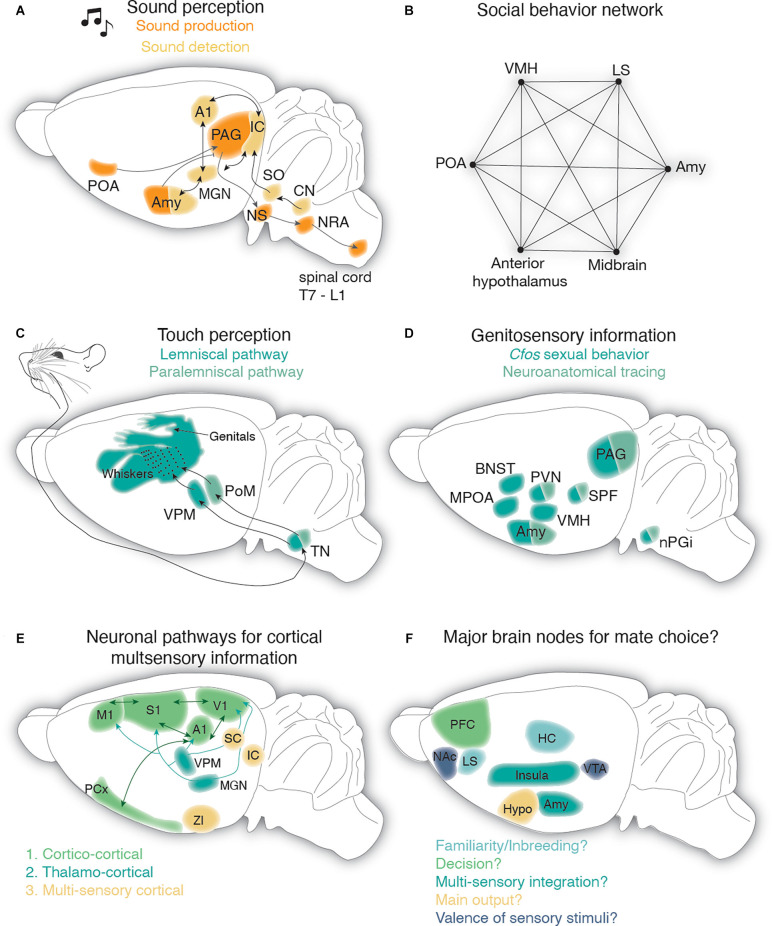
Neural circuits for audition and somatosensation in the context of rodent mate choice. **(A)** Sound detection pathway is depicted in light yellow: auditory information from the cochlea is transmitted *via* the cochlear nerve to the cochlear nucleus (CN) which sends information to the superior olive from where it reaches the inferior colliculus. From the inferior colliculus, auditory information reaches the auditory cortex (A1) or amygdala (Amy) *via* the medial geniculate nucleus in the thalamus (MGN). IC and MGN are reciprocally connected with A1. Reciprocal connection between MGN and Amy has been observed as well. Sound production pathway is highlighted in orange: the periaqueductal gray (PAG) has been involved in mate choice produced USVs and receives auditory related feedback from the preoptic area (POA) and Amy. From the PAG information is sent to vocal pattern generators in the brain stem (nucleus solitarius, NS and nucleus retroambiguus, NRA) and from there is further processed in the spinal cord. **(B)** The social behavior network as postulated by Newman (1999) consists of reciprocal connections between the ventromedial hypothalamus (VMH), the later septum (LS), the amygdala (Amy), the midbrain, the anterior hypothalamus, and the preoptic area (POA). **(C)** Touch vibrissae information travels from the periphery through the trigeminal nucleus (TN) in the brainstem to the thalamus. In the thalamus, information is relayed *via* the ventral posteromedial (VPM) and the medial posterior complex (POm) nuclei to the vibrissae portion of the primary somatosensory cortex (S1): the barrel cortex. **(D)** Genitosensory information has been shown to activate various subcortical structures, including brain areas of the social behavior network, as revealed by cfos studies in male and female rats. Ascending genitosensory encoding information from the penis and vagina in line with unraveling cfos activitiy during sexual behavior revealed the nucleus paragigantocellularis (nPGi), the subparafascicular nucleus (SPF), PAG, periventricular nucleus of the hypothalamus (PVN), and Amygdala (Amy). BNST, bed nucleus of stria terminals; MPOA, medial preoptic area. **(E)** Potential pathways leading to multisensory processing in primary cortical areas (M1, primary motor cortex; S1, primary somatosensory cortex; A1, primary auditory cortex; V1, primary visual cortex; PCx, piriform cortex): cortico-cortical connections are depicted in dark green. Thalamocortical connections carrying multimodal information are drawn in turquoise (VPM, ventral posteromedial thalamus; MGN, medial geniculate nucleus) and connections from multisensory integration centers are shown in light yellow (ZI, zona incerta; IC, inferior colliculus; SC, superior colliculus). **(F)** Postulated major brain nodes implicated during mate choice. Prefrontal cortex (PFC) may account for the active decision to mate. The lateral septum (LS) and hippocampus (HC) might be responsible for individual and (non)familiar recognition of potential mates. The Insula and amygdala (Amy) are proposed to be the main integrators for multimodal information. The hypothalamus (Hypo) is most likely an active output player during mate choice as it has been described during other socio-sexual behaviors. The reward pathway of the ventral tegmental area (VTA) and nucleus accumbens (NAc) encodes the valence of sensory stimuli during mate choice.

While a large body of literature exists describing the neural circuitry involved in USV production (Hernandez-Miranda and Birchmeier, 2018), processing, and detection (Jürgens, 2002; Pickles, 2015), our knowledge of whether auditory signals are differentially processed in the context of social behavior or in particular mate choice is rather limited. Briefly, auditory information is transmitted from the cochlea *via* the auditory nerve to the brainstem. Within the brainstem auditory information is transmitted from the cochlear nucleus (CN) to the superior olivary nucleus (SO) and from there to the midbrain, namely to the inferior colliculus (IC; Figure 3A). The IC sends information further to the thalamic relay station (medial geniculate body) from where information reaches the primary auditory cortex (A1; Asaba et al., 2014) and the amygdala (LeDoux et al., 1985, Ledoux et al., 1987; Doron and Ledoux, 2000; Ferrara et al., 2017) and this has recently been confirmed for mice (Keifer et al., 2015; Lohse et al., 2021). While it is clear that mice USVs with their broad frequency range are differentially processed in IC (Portfors et al., 2009; Wooley and Portfors, 2013; Garcia-Lazaro et al., 2015) and even already in CN (Roberts and Portfors, 2015), few studies have investigated socio-sexual modulation of USV responses in these brain stem nuclei of male and female (Hanson and Hurley, 2014; Keesom and Hurley, 2016). Female mice premate choice behavior is reflected in the IC serotonergic levels of male mice (Keesom and Hurley, 2016) with IC serotonin measures increasing upon female acceptance and decreasing when the female rejects the male. Whether the reflection of female acceptance or rejection is encoded by the ensemble of auditory, visual and somatosensory stimuli, since multimodal processing has been described in the IC (Gruters and Groh, 2012; Yang et al., 2020) and CN (Young et al., 1995; Kanold and Young, 2001; Shore, 2005), remains unclear but might be shaped by modulatory input (Hurley et al., 2002; Nevue et al., 2016; Beebe et al., 2021) or reciprocal connection to A1 (Blackwell et al., 2020). Audible female sounds (squeaks) seem to be reflected in the activity of male IC neurons as shown by anesthetized single cell recordings in mice (Gentile Polese et al., 2021).

Regarding sound production (Figure 3A), a recent rat study unraveled distinct nuclei in the brain stem acting as a vocal pattern generator, segregated by the USVs emotional meaning. While 50 kHz pleasurable calls are generated by the parvocellular reticular formation, 20 kHz alarm calls seem to be produced by a posterior region, mapping to the nucleus retroambiguus (Hartmann and Brecht, 2020). Whether these centers receive differential inputs from the mesolimbic dopaminergic system (shown to initiate 50 kHz calls; Burgdorf et al., 2007; Ciucci et al., 2009; Willuhn et al., 2014) and cholinergic systems (responsible for 22 kHz; Brudzynski, 2021) thereby driving different USVs during mate choice, such as 50 kHz during premating/mating and 20 kHz during sexual satiety remains to be investigated. In fact, a recent mouse study found that the nucleus retroambiguus receives its main input from upstream neurons in the periaqueductal gray (PAG) that were strongly involved in USV production in the presence of a female (Tschida et al., 2019). Even though the latter study carefully disentangles a PAG to hindbrain vocal circuit in the context of mate choice, showing that female affiliative behaviors towards the male are clearly reduced when male calling is prevented, it is still unknown how male USVs shape female preference at the neural circuit level. In general, studies in female rats (Sakuma and Pfaff, 1979; Yamada and Kawata, 2014) and mice (Ishii et al., 2017) point to a lordosis modulating role of the PAG supported by anatomical studies that have uncovered various PAG-brainstem projections that are activated during mating (Yamada and Kawata, 2014; Subramanian et al., 2018; Lo et al., 2019).

Auditory cortical responses to natural USVs have been studied in mice (Chong et al., 2020; Maor et al., 2020; Royer et al., 2021) and rats (Carruthers et al., 2013; Kim and Bao, 2013; Rao et al., 2014; Bao, 2015), but not in a mating context. Potential multimodal processing in A1 has been best described in the context of maternal behavior by testing the synergistic action of pup odors and USVs (Cohen et al., 2011; Cohen and Mizrahi, 2015; Tasaka et al., 2018, 2020; Nowlan et al., 2022). Regarding mate choice or sex-representation, rat A1 activity seems to be differentially modulated by the opposite sex (Ebbesen et al., 2019).

Auditory information reaches the amygdala, belonging to the social brain network, *via* the thalamus. The social brain network (Figure 3B) is composed of six reciprocally connected brain areas, the amygdala, the lateral septum, the preoptic area (POA), the anterior hypothalamus, the ventral hypothalamus, and the midbrain (Newman, 1999), and is involved in various socio-sexual behaviors. The amygdala has been described in the context of fear conditioning during sound perception (McCue et al., 2014; Cragg et al., 2016) and processing (Sadananda et al., 2008; Ouda et al., 2016; for a detailed overview see Furtak and Brown, 2018). Moreover, single basolateral amygdala neurons in the male rat are not only activated by female USVs (Grimsley et al., 2013) but also seem to distinguish between different contexts and the type of call (Parsana et al., 2012; Schönfeld et al., 2020). In male mice, the amygdala has been recently attributed to the sound-producing pathway, as medial-central amygdalar GABAergic neurons directly inhibit USV-producing neurons in the PAG, while other aspects of socio-sexual behaviors were not affected (Michael et al., 2020). Inhibitory neurons from the POA on the contrary disinhibit PAG USV-producing neurons, suggesting that the amygdalar PAG pathway suppresses USV production in male mice, whereas the POA-PAG pathway favors USV production (Michael et al., 2020) and modulates the mate contextual male calls (Chen et al., 2021) which could be gated by different incoming female sensory cues probably relayed from interconnectivity with the amygdala (Newman, 1999). The amygdala is not only included in the auditory pathway but has been probably best described within the olfactory pathway (Mucignat-Caretta, 2021), which is the main player in sexual approach behavior (Bergan et al., 2014; Li et al., 2017; Lenschow and Lima, 2020). Moreover, it has access to general tactile information (Shi and Cassell, 1999) and genital somatosensory cues (Erskine, 1993; Oberlander and Erskine, 2008). Altogether this points to the amygdala as a multimodal computational brain node (Figure 3E).

### The primary somatosensory cortex and socio-sexual touch

Peripheral tactile information sensed by the whiskers, genitals, and other body parts (Huzard et al., 2022) is relayed *via* ventral medial and posterior medial thalamic nuclei before reaching and being processed in the primary somatosensory cortex (S1, Figure 3C) that contains a somatotopy of the outer body (reviewed in Adibi, 2019). The impact of vibrissae S1 (barrel cortex) activity during socio-sexual behavior has been described during play behavior (Gordon et al., 2002; Charles Lawrence et al., 2008) and social facial touch (Bobrov et al., 2014; Lenschow and Brecht, 2015; Clemens et al., 2019). At least 40% of barrel cortex neurons are differentially modulated by social facial touch compared to object touch. Moreover, the female’s barrel cortex activity is modulated with the estrous cycle as single unit recordings increase their firing when estrous females touch a male but are inhibited when a female conspecific is touched whereas out of estrus this phenomenon was absent (Bobrov et al., 2014).

Sensory genital stimulation leads to stronger activity in the male genital portion of S1 than in the female genital cortex of S1 and this has been shown for rats (Lenschow et al., 2016) and mice (Sigl-Glöckner et al., 2019). Interestingly an early study on the rat shows an increase in the cortical genital sensory receptive field during estrous (Adler et al., 1977). To what extent rat and mice S1 genital cortex of males and females differentially respond to anogenital sniffing in a mate choice paradigm or the mating context remains open.

### Multisensory integration in primary cortical areas

All these studies point to a certain degree of multisensory integration in S1 as the neuronal activity does not reflect the mere tactile input but also the interaction partner’s sex as S1 barrel cortex activity of female rats is differentially modulated by female vs. male interaction partners (Bobrov et al., 2014). Indeed, multisensory integration of social facial touch and USVs has been described in rat A1 (Rao et al., 2014). While the authors report a striking inhibition when aligning single A1 units to facial touch, a strong modulation of auditory cortex neurons to USVs by facial touch could be seen (Rao et al., 2014) indicating a multimodal processing occurring during a social context. In a follow-up study the authors could show that A1 seems to process sex-touch responses as well leaving it unclear if the mechanism behind this phenomenon might be of neuromodulatory origin. The same was observed in the primary vibrissae motor cortex, cingulate cortex, and prelimbic cortex (Ebbesen et al., 2019).

Touch and odor processing has been recently described in S1; although not in the context of mate choice (Renard et al., 2021).

The multisensory effect on the cortical level could be inherited from subcortical inputs (Figure 3E): indeed major thalamic auditory (the medial and dorsal regions of the medial geniculate body respond to visual, somatosensory, and vestibular inputs; Wepsic, 1966; Calford and Aitkin, 1983; Komura et al., 2005) and somatosensory relays (ventromedial posterior nucleus combines tactile and visual cues; Bieler et al., 2018; Lohse et al., 2021) have been shown to be involved in multisensory integration and to act as a context-dependent gate favoring one modality vs. the other (Lohse et al., 2021). Another pathway that could give rise to multimodal signals in early cortical areas is through cortico-cortical reciprocal connections like as shown between S1, A1, and primary visual cortex (Budinger et al., 2006; Stehberg et al., 2014; Meijer et al., 2019). The perirhinal cortex has been of long-standing interest to transmit odor information to primary sensory cortical areas (Winters and Reid, 2010; Albasser et al., 2011; Renard et al., 2021). Lastly, cortical-multi-sensory processing might stem from indirect afferent inputs of classical multisensory computational cores, like the superior colliculus (Ahmadlou et al., 2018; Gharaei et al., 2020; Benavidez et al., 2021). In fact, the genital cortex of S1 receives scarce but substantial input from the zona incerta (Lenschow and Brecht, 2018; Massé et al., 2019), a so far neglected brain area, recently described as a potential relay for multisensory integration (Wang et al., 2020).

### Neural processing of audition and touch in the social behavior network

While impressive progress has been made in disentangling specific cell activity during sexual behavior in the social behavior network structures (Lenschow and Lima, 2020) our knowledge of how these brain areas encode audition and touch information is scarce and this is, in particular, true in the context of mate choice. Using the immediate early gene *cfos* as a readout of neural activity, various studies in female rats (Erskine, 1993; Erskine and Hanrahan, 2003; Oberlander and Erskine, 2008) found an activation of the nucleus paragigantocellularis (nPGi), medial preoptic area (MPOA), bed nucleus of the stria terminalis (BNST), PAG, ventromedial hypothalamus (VMH), and Amygdala (Amy) during artificial or natural (through male mounts with intromissions) clitoral and vaginocervical stimulation. Likewise *cfos* induction upon copulation has been found in the nPGi, MPOA, BNST, Amy and subparafascicular nucleus of the thalamus (SPF) in male rats (Paredes and Baum, 1995; Coolen et al., 1998). Anatomical mapping of ascending genitosensory information in male (Gréco et al., 1996, 1998; Normandin and Murphy, 2011) and female rats (Marson and Murphy, 2006; Gelez et al., 2010) revealed targets consistent with the aforementioned cfos studies. The study by Normandin and Murphy (2011), however, unraveled that only the nPGi, PAG, SPF, and PVN were labeled with a penis/vagina injected anterograde traveling herpes virus in parallel with cfos induction upon sexual behavior (Figure 3D).

Even though the aforementioned literature suggests that genitosensory information may reach the social behavior network, we are still lacking a fine description of the pathways carrying somatosensory and auditory information to the social behavior nodes and how sensory information coding during mate choice may differ from other social contexts, such as parenting or aggression.

### Mate choice decision

Even though multimodal processing seems to be a common feature in early auditory and somatosensory cortical areas and these signals may be inherited from thalamic or multisensory centers (Figure 3D), there must be brain structures that have access to multi-modal cue processing and actively guide the mate choice decision during premating, mating and even postmating and these areas should be connected to the social behavior network (Figure 3C); more specifically to the amygdala that has been proposed to be the main integrator for multisensory cues (Raam and Hong, 2021) and to the hypothalamus, shown to be the main output player during various socio-sexual behaviors (Wei et al., 2021). Both areas consist of multiple substructures or nuclei highly inter- and reciprocally connected to other social behavior network nodes, thereby able to actively contribute to social cognition (Chen and Hong, 2018), the mechanism of “acquiring, processing, keeping and reacting on social information” (Seyfarth and Cheney, 2015; Kavaliers and Choleris, 2017).

Thinking of cognition, and in particular, decision making, the prefrontal cortex (PFC) might be the most obvious player coming into the equation. Various recent studies investigated PFC activity during active decision making in the mouse (Bicks et al., 2015; Vertechi et al., 2020; Posner et al., 2022) and rat (Kurikawa et al., 2018; Verharen et al., 2020) describing multi-modal processing capacities (Bizley et al., 2016; Shadi et al., 2020; Coen et al., 2021; Zheng et al., 2021) and implications during social cognition (Yizhar et al., 2011; Kumar et al., 2014; Felix-Ortiz et al., 2016; Murugan et al., 2017; Levy et al., 2019; Mague et al., 2020). More particular, multiple studies point to a prominent role of PFC during opposite-sex choice and social approach behavior (Nakajima et al., 2014; Kim et al., 2015; Lee et al., 2016; Jennings et al., 2019; Levy et al., 2019; Kingsbury et al., 2020). Female PFC oxytocin receptor expressing neurons in particular seem to be crucial for male preference during sexual receptivity as their ablation abolishes male approach behavior (Nakajima et al., 2014). Microendoscopic imaging revealed that dorsomedial PFC neurons predict the opposite partner preference as their activity reflected female-preferring choices but not when a male mouse was the preferred partner (Kingsbury et al., 2020). Moreover, a direct connection from the PFC to the lateral septum (LS) has been described in the same study. By specifically activating a genetically defined LS projecting cell population in the PFC the authors showed that mice preferred to interact with non-familiar mice. This connection could be a potential top down control on how the cortex impinges on the social behavior network thereby driving a preference. A similar circuit has been described during social dominance behavior (Padilla-Coreano et al., 2022). Whether this is mediated by the integration of multisensory cues has been left open.

Sensory information in the mPFC encoding the sex and social valence of a conspecific might stem from the hippocampus (Murugan et al., 2017). The dorsal CA2 and ventral part of the hippocampus have been tremendously studied in the light of social recognition and memory (for review see Okuyama, 2018; Wang and Zhan, 2022). As mentioned earlier the latter is of utmost importance during premating choice in order to prevent inbreeding or to mate with the same partner. In fact, through immediate early gene-based connectivity in mice, it was shown that protein synthesis in the hippocampus, but also mPFC and amygdala mediates conspecific memory (Ferretti et al., 2019). Interestingly, ventral CA1 neurons are strongly modulated by touch and USV calls during rat social interactions. In male rats, ventral CA1 cells showed stronger responses toward females than to males and more interestingly seem to distinguish between individual females independent of their estrous phase. Which sensory modality drove this individual female recognition was however not pinned down (Rao et al., 2019). Septal-hippocampal connections has been described in rats (Risold and Swanson, 1996, 1997; Arszovszki et al., 2014) and mice (Parfitt et al., 2017; Leroy et al., 2018; Horiai et al., 2020) and the recent description of encoding kinship behavior in the LS (Clemens et al., 2020) suggests that these two areas might be key to identify the partners individuality and prevent repeated sex within the same partner (Figure 3F). Both structures have not been investigated in the context of mate choice but the hippocampus has been described in USV production (Sprouse and Aghajanian, 1988; Antoniadis and McDonald, 2000; Wöhr et al., 2009; Hamed et al., 2016; Huang et al., 2020) and detection (Ouda et al., 2016), although it is unclear whether hippocampal neurons differentiate sex, familiarity, or individuality based on USVs. It is however noteworthy that ventral CA1 neurons distinguish between the calls from others and their own call (Rao et al., 2019). Neurons in the LS are modulated by USVs even though no discrimination is made between kins or non-kins (Clemens et al., 2020) and cholinergic stimulation triggers calls in the lateral septum of anesthetized rats (Brudzynski, 2021). Studies inspired by Clemens et al. (2020) and Rao et al. (2019) should be conducted in the mating context in order to unravel the hippocampal-septal interconnectivity and their implementation towards the prevention of inbreeding (in mice) or remating with the same animal by using multi-modal cues.

Prefrontal-amygdala connections have been shown to be involved in social-decision making (Gangopadhyay et al., 2021) and more specifically to drive social approach/preference behavior (Huang et al., 2020; Kuga et al., 2021) through positive vs. negative valence-encoding (Felix-Ortiz et al., 2016; Huang et al., 2020). There are also recent studies pointing to the PFC (Zhou et al., 2017; Padilla-Coreano et al., 2022) and amygdala (Hong et al., 2014; Lee et al., 2016, 2021; Dwortz et al., 2022) as neural correlates for social hierarchy as the dominance level, for instance, was reflected in the gene expression of corticotropin releasing factor (So et al., 2015) in the medial amygdala. Whether neuronal activity in the PFC and amygdala predicting social hierarchy and rank are also reflecting differential processing for USVs or vibrissae is currently unknown.

### The insula as a bridge between primary sensory cortices and the social behavior network

While the amygdala seems to be the central multisensory node in the social behavior network, early cortical areas, the main drivers for early sensory processing barely connect to the amygdala or the social behavior network in general. Therefore, the insula has been proposed to be a prominent bridge (Rogers-Carter and Christianson, 2019) between the social behavior network and sensory cortical and thalamic nodes (Figure 3F). A recent study examined the inhibitory and excitatory afferents and efferents of the mouse insula (Gehrlach et al., 2020) thereby supporting awareness of a unique position to receive and process multimodal cues (Rodgers et al., 2008; Gogolla et al., 2014) and driving emotional and socio-cognitive decisions (Gehrlach et al., 2020). A recent mice study described that 24% of agranular insular neurons encode social exploration and distinguish between anogenital, nose-to-nose, or body exploration (Miura et al., 2020). Insula-amygdala interconnectivity (Shi and Cassell, 1999; Gehrlach et al., 2020) for instance has to be shown to be crucially involved in positive and negative valence coding during social interactions (Ferretti et al., 2019; Nicolas et al., 2021) such as pathogen avoidance (Kavaliers et al., 2022). Intriguingly insula function may be decisive to discriminate between sick and healthy rats (Rieger et al., 2022) whereas the amygdala seems to have a preventive but not a causal role (Kwon et al., 2021) in signaling sickness and overall sociability. Affective touch during social encounters has been shown to modulate insula activity in rats (Rogers-Carter et al., 2018; Miura et al., 2020) and mice (Miura et al., 2020; Matsumoto et al., 2021) while there are no studies evaluating insular modulation by social vocalizations or USV in general. Even though multimodal processing in the insula has been described in the rat and mouse (Rodgers et al., 2008; Gogolla et al., 2014) and causal insular impact during social cognition (Rogers-Carter et al., 2018; Rogers-Carter and Christianson, 2019), such as novelty recognition (Kim et al., 2021) has been demonstrated, mate choice studies measuring insula activity during the different phases are missing.

### Neural pathways underlying socio-sexual valence of mate choice

The reward pathway, consistent with the ventral tegmental area (VTA) and the nucleus accumbens (NAc; Russo and Nestler, 2013), play an important role in evaluating the valence of sensory stimuli during mate choice (Figure 3F; Beny-Shefer et al., 2017; Hu et al., 2021; Sun et al., 2021) and social interactions in general (Bariselli et al., 2018; Solié et al., 2022) mostly through reciprocal connectivity with the amygdala, hypothalamus, and prefrontal as direct inhibition of dopamine (DA) VTA neurons do not alter the facilitation of sexual behavior (Beloate et al., 2016). Many studies emphasized that a paced mating is needed in order to induce a reward state ensuring the maintenance and repetition of the behavior (reviewed in Camacho et al., 2009). In line with this it could be shown that DA levels rise in the nucleus accumbens and VTA during paced mating in female rats (Mermelstein and Becker, 1995; Pfaus et al., 1995; Becker et al., 2001) whereas in two studies (Mermelstein and Becker, 1995; Pfaus et al., 1995) that DA rise was already observed before the actual paced mating took place, leaving it unclear if the latter was due to smell, vision, or USVs or an ensemble of all. The extent to which DA is indeed a reward signal resulting from sexual stimulation has been challenged by pharmacological studies in rats which failed to induce a conditioned partner preference after the application of a DA antagonist (for an in-depth opinion see Paredes, 2009). Recent mice studies making use of DA sensors (Dai et al., 2021) in combination with cellular specificity and high temporal resolution tools (Beny-Shefer et al., 2017), however, rather favor an anticipatory role of DA during sexual stimulation and a clear necessity of DA transmission in the mesolimbic pathway during male mate choice (Beny-Shefer et al., 2017).

Microdialysis in the basolateral amygdala (a projection target of the VTA) while presenting male and female mice with USVs from the opposite sex showed an increase in DA levels in both males and females (Ghasemahmad, 2020), indicating the overall incentive rewarding nature of USVs. There are also a few studies investigating the rewarding neural correlate of social touch (Sun et al., 2018; Elias and Abdus-Saboor, 2022). Intriguingly, by artificially activating a specific sensory cell population encoding the transmission of gentle stroking they could trigger the lordosis state in female mice independent of their reproductive cycle (when the back was targeted). Moreover, the authors could show that DA is released in the NAc when optogenetically activating these sensory cells in the back of the female (Elias and Abdus-Saboor, 2022). Another study making use of dopamine sensors in the NAc observed DA transients during male mice sexual behavior and even though the authors do not disentangle different sensory modalities, body contact during mounts with penile thrusting led to DA increase in the NAc (Sun et al., 2018). Whether the rewarding valence of social touch reaches the reward pathway through primary cortical to subcortical circuits (Lenschow and Brecht, 2018) or rather by a bottom-up pathway (from the spinal cord through the PAG and hypothalamus; Figure 3D; Elias and Abdus-Saboor, 2022) demands further investigation. Lesion studies of the MPOA in male (Paredes et al., 1998) and female rats (Meerts and Clark, 2009) are in favor of the latter as conditioned partner/place preference upon vaginocervical stimulation and paced mating are disrupted indicating that the MPOA is an important site for mating associated processing of somatosensory signals and the reinforcing effect of vaginocervical stimulation (Meerts and Clark, 2009).

Taking into consideration all above-mentioned studies examining neural activity during socio-sexual behaviors, it gets clear that we have a great gasp on the main brain nodes being involved during social evaluation and approach behavior, even though with no or very little conclusions to draw in the context of USV and touch processing. Whether the neuronal activity in the amygdala, hypothalamus, hippocampus, lateral septum, prefrontal cortex, or reward pathway during mating and postmating might predict the reproductive outcome or could even predict an on-going or postmating choice demands further experiments taking advantage of natural settings (Krakauer et al., 2017) and state-of-the-art neural recording and manipulation techniques.

## Neural Circuits for Audition and Somatosensation in The Context of Human Mate Choice

### Somatosensation

**Figure 4:**
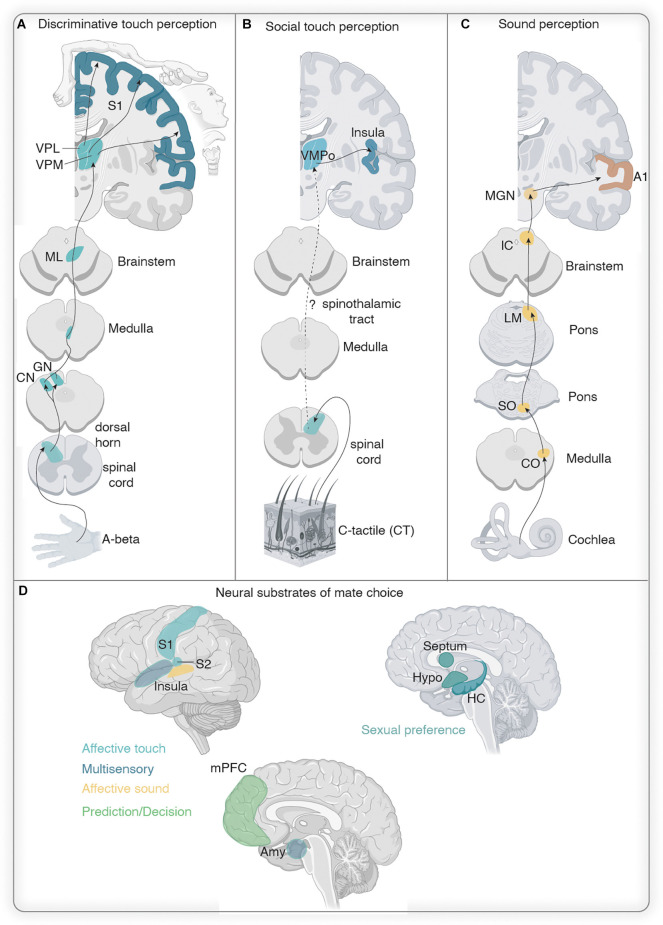
Neural circuits for audition and somatosensation in the context of human mate choice. **(A)** Discriminative touch is thought to be relayed to the dorsal spinal cord *via* A-beta fibers primarily found in glabrous skin. Touch information is then sent to the cutaneous nucleus (CN) and gracilis nucleus (GN) and travels further through the medulla and mediolateral brainstem before being relayed by the thalamus (ventral posterolateral, VPL, and ventral posteromedial, VPM nuclei) towards the primary somatosensory cortex (S1) which contains a topography of the outer body, known as the homunculus. **(B)** Social touch is predominantly transmitted *via* C-tactile (CT) fibers to the dorsal spinal cord. The ascending route through the medulla and brainstem is not unraveled. Social touch is thought to be primarily relayed *via* the ventromedial part of the posterior thalamus (VMPo) towards the insular cortex. **(C)** The sound perception pathway. Auditory information is perceived by the cochlear which transmits the information *via* the cochlear nerve to the cochlear nucleus (CN) from where is sent to the superior olive (SO) and further through the nucleus of the lateral lemniscus (LM) to the inferior colliculus (IC) in the brainstem. The medial geniculate nucleus (MGN) is the thalamic relay station from which information is sent to the primary auditory cortex (A1). **(D)** Potential neural substrates of human mate choice. Brain, spinal cord, hand, skin, andcochlea icons were created with biorender.

Mechanoreceptive afferents in the skin, such as fast myelinated A-beta or slow conducting C-tactile (CT) fibers, are activated upon touch and CT-fibers have been predominantly found in hairy skin (Figure 4A) whereas A-beta is prevalently present in glabrous skin like the hand (Figure 4B; McGlone et al., 2014). While the latter are involved in many tactile attributes (Mountcastle, 2005; Kandel, 2013), CT-fibers are mainly activated by slow stroking of the skin, as is observed during caresses. Due to the differential activation by velocities (Essick et al., 1999; Vallbo et al., 1999, 2016; Löken et al., 2009) and supposedly different integrative neural pathways (McGlone et al., 2014; Morrison, 2016; Case et al., 2016; Marshall and McGlone, 2020), a functional dissociation has been suggested (McGlone et al., 2014): A-beta fast conducting afferents are rather involved in discriminative touch (Figure 4A), while the CT-fibers may signal affective or social touch (Figure 4B).

Various human fMRI studies examining discriminative touch mainly delivered to the hand reported activity in the posterior column medial lemniscal pathway (Hansson and Brismar, 1999; Blatow et al., 2007; Govers et al., 2007; Agosta et al., 2009; Ghazni et al., 2010) which is preserved across mammals. Briefly tactile information from the skin is transmitted to the dorsal region of the spinal cord from where it reaches the gracilis (GN) and cutaneous nucleus (CN) in the medulla. Afferents then cross the midline and travel through the medial lemniscus in the brainstem to the ventral-posterior lateral (VPL) and medial nuclei (VPM) in the thalamus from where they reach S1 containing a topography of the body also known as homunculus (Penfield and Rasmussen, 1950; Figure 4B). For a recent review on somatosensory processing please refer to de Haan and Dijkerman (2020).

The social touch pathway, recently debated (Marshall et al., 2019), supposedly travels through the spinothalamic tract (Foerster and Breslau, 1932; Lahuerta et al., 1994) and ventromedial posterior nucleus (VMPo) before entering the insula (reviewed in: Marshall and McGlone, 2020). Even though neurophysiological and neuroimaging studies support separated neuronal processing of discriminative (somatosensory cortices activation) vs. affective touch (insula and orbitofrontal cortices activation; Francis et al., 1999; Olausson et al., 2002; Ebisch, 2011; McGlone et al., 2012; Gordon et al., 2013; Case et al., 2016; Morrison, 2016; Kirsch et al., 2020), there is a broad body of contradicting studies reporting pleasurable touch by both hairy and glabrous skin (Luong et al., 2017; Pawling et al., 2017; Schirmer and Gunter, 2017) and primary sensory areas also being activated by affective touch (Gazzola et al., 2012; Ellingsen et al., 2013; Ebisch et al., 2014a; Shirato et al., 2018; Schirmer et al., 2022). A recent fMRI study adds to this contradiction as partner vs. stranger touch led to stronger activation of the orbitofrontal cortex, posterior cingulate cortex, and somatosensory cortices but not the insula (Kreuder et al., 2017) which however showed an increased activity upon touch when oxytocin was nasally applied. Oxytocin, in general, was suggested to play a role in pair bonding in humans and is indeed released during sexual intercourse (Winslow et al., 1993; Uvnäs-Moberg et al., 2005) but also non-sexual contact in long-term couples (Shermer, 2004; Light et al., 2005). Strikingly, a recent study, although not investigating the role of oxytocin, found that holding hands with the love partner induced higher interpersonal neural synchronization than vocal communication and was absent during interpersonal friend touch (Long et al., 2021).

Insula activity modulations during social touch by romantic partners were related to sexual desire and the expected outcome (Ebisch et al., 2014a, b). Integration of social touch with other sensory modalities has been described to alter insula activity, as unpleasant odors (Croy et al., 2016) or fearful context (Koole et al., 2014) hampers its responses.

Despite cortical and insula activation upon partner’s touch, prominent amygdala activation has been reported when being or thought to be touched by the partner (Suvilehto et al., 2021). Interestingly, the amygdala shows also strong fMRI responses during visual guided short-term mate choice (Turk et al., 2004; Funayama et al., 2012; Cartmell et al., 2014) and in particular in humans that have seen pictures of their love partners (Bartels and Zeki, 2000, 2004; Aron et al., 2005; Fisher et al., 2005).

As human courtship progresses, tactile cues gain sexual weight and more erogenous zones, such as the genitals, are touched. Genital sexual stimulation by partners leads to strong activation of S1 but also the insula (Georgiadis et al., 2009; Chivers et al., 2010) whereas the amygdala was more strongly activated in women than in men (Georgiadis et al., 2009). Insula genital touch activation is intriguing in the light of discriminative vs. affective touch as it is to date unknown whether CT fibers can be found in the genitals (Georgiadis and Kringelbach, 2012). Interestingly a recent human fMRI study described that the somatosensory region representing the clitoris is larger in women who reported to have higher frequencies of sexual intercourse in the past year and during the onset of their sexual activity (Knop et al., 2022).

For a detailed review regarding brain activation during sexual intercourse, please refer to Ruesink and Georgiadis (2017) and Calabrò et al. (2019).

### Audition

Just like the touch perception pathway, the auditory detection pathway is preserved across rodents and humans (Figure 4C), with specific auditory areas being sensitive to voices (Belin et al., 2000, 2002; Lattner et al., 2005; Zaehle et al., 2008; Allen et al., 2017). Human fMRI activity in A1 of men can decode the sex from voice (Sokhi et al., 2005) and even distinguish between individuals (Andics et al., 2010). Moreover, A1 seems to encode vocal emotions as shown by various fMRi studies (Kotz et al., 2003; Grandjean et al., 2005; Ethofer et al., 2006, 2007, Ethofer et al., 2009; Wiethoff et al., 2008). Other areas involved in encoding vocal emotions and gender information are the prefrontal cortex, amygdale, and insula (Ross, 1981; Heilman et al., 1984; George et al., 1996; Morris et al., 1999; Buchanan et al., 2000; Sander and Scheich, 2001; Wildgruber et al., 2002; Mitchell et al., 2003; Junger et al., 2013; Kotz et al., 2013), all areas that have been shown to be activated by tactile mating stimuli.

### Mate choice prediction

The human prefrontal cortex has been implicated in executive functions and decision-making (Domenech and Koechlin, 2015). Thus, not surprisingly it has been revealed as another prominent decisive and consistent activated area during short-term and long-term mate choice (Bartels and Zeki, 2000, 2004; Turk et al., 2004; Fisher et al., 2005, 2006; Cooper et al., 2012; Cartmell et al., 2014; Ueda et al., 2017, 2018).

Interestingly, a recent study using functional near-infrared spectroscopy in a speed dating paradigm revealed that interpersonal synchronization activity of the dorsolateral prefrontal cortex predicted mate preference (Yuan et al., 2022) just as it could be described for mice (Kingsbury et al., 2019, 2020). On what sensory stimuli this prediction was based is unclear, but the preference was mainly formed by social rather than physical attractiveness indicating a multimodal action of cues, like facial expression, pleasant voice, and smell. However, interpersonal synchronization has been also described to be correlated with partner-oriented kissing satisfaction during which tactile stimuli play the main role (Müller and Lindenberger, 2014). In line with the latter, encoding of socio-sexual touch by prefrontal areas has been shown as well (Rolls et al., 2003; Mccabe et al., 2008).

### Neural pathways for sexual preference/wanting sex

Intriguingly none of the above studies examining human brain activation by affective touch or voice in the mate choice context pinpointed hypothalamic, septal, or hippocampal activity, even though these areas have been shown to be differentially modulated by the opposite sex in rats and mice. This might be in part because most of the reviewed literature only used simple pictures or cues delivered by their love partners or to neutral body parts that do not trigger a sexual preference. A recent meta analytic review reported that when sexual preference was the center of interest, such as that participants needed to value whether they would want sex or could imagine reaching an orgasm with a stranger, a preserved neural circuit consisting of hypothalamic, septal, and hippocampal region was activated (Poeppl et al., 2016). What we conclude from this is that hypothalamic, septal, and hippocampal regions could be considered as the key player when sex is the desired outcome and that prefrontal, cortical, insula, and amygdala regions are mainly involved as soon as mate choice happens in a socio-emotional context (Figure 4D). Various fMRI and PET studies undermine this hypothesis as hypothalamic, hippocampus, and septal activation has been shown to be activated during sexual preference, sexual arousal, and intercourse (reviewed in Ruesink and Georgiadis, 2017; Calabrò et al., 2019).

All of the above-mentioned literature is based on human fMRI, PET, lesion, or EEG studies (Poeppl et al., 2016, reviewed in Ruesink and Georgiadis, 2017; Calabrò et al., 2019) and despite the obvious caveats in comparison to high-resolution recording methods in rodents, the biggest drawback might be the lack of conclusion about interconnectivity and direction of information processing. Nevertheless, it is noteworthy that the interplay of the prefrontal cortex, septum, hippocampus, amygdale, and hypothalamus just as in rodents underlies human socio-sexual processing (Figure 4D) and that some of these nodes have been observed to be directly modulated in a mate choice context.

## Conclusions and Outlook

Mate choice as a potent generator of diversity and a fundamental pillar for sexual selection and evolution has been the focus of intensive study since Darwin. Mate choice requires numerous stages of behavioral and neural processing, initiated by the identification of a conspecific, which will be further scrutinized to evaluate his/her potential to sire progeny. The evaluation can occur at any stage during premating, mating, and postmating behavior, and decisions can be reversed at almost any point. Although the weight that each sensory modality contributes to mate assessment is species-dependent, it is long acknowledged that the information provided by a mate is multimodal in nature: while the initial detection is often dominated by a single modality, other senses can offer various information that must be equally evaluated once the interaction develops. Interestingly, the processing of multimodal cues can work in distinct ways, as in some situations different cues are redundant and synergize, while in others they take divergent meanings and compete.

Our understanding of the neural mechanisms underlying mate choice has been driven in part by rodent and human studies during which smell and vision have received considerable attention. This is not only due to these modalities being the most dominant for these species but also because of existing immense knowledge regarding the neural pathways underlying their information processing and their guidance of non-social behaviors. In stark contrast, very little is known about how information from various senses, in particular audition and touch, is processed and interacts with olfactory and visual cues. Multisensory integration has gained substantial attention in the past decade and the number of brain regions processing multimodal cues during social and non-social behavior has grown. Given the sensory complexity of mate choice at all stages, multi-sensory integration will most certainly also be unraveled during that behavior. Moreover, how the internal state, e.g., sexual arousal, affects such processing has received no investigatory attention so far.

The fact of no existent well delineated “circuit for mate choice” makes the research of outlined questions even more challenging. Nevertheless, strides have been made to fill the gap in knowledge and, given the social nature of mate choice, it is not surprising that some of the regions involved in processing mate choice cues overlap with the so-called social brain network, known to impact other social behavior such as parenting and aggression. Simultaneous multi-area recordings and activity manipulations of specific cell populations will be key to understand: (i) whether the circuits underlying mate choice will overlap with social behavior networks; (ii) how the latter are connected to higher cognitive brain regions involved in decision making; and (iii) whether brain nodes described as classical multisensory integrators are at the interface between those two.

## Author Contributions

CL outlined the topic of review and structured it. CL and AM did literature research. CL, AM, and SL wrote the manuscript. All authors contributed to the article and approved the submitted version.

## Funding

CL is supported by a Human Frontier Science Program Postdoctoral Fellowship (LT000353/2018-L4) and H2020 Marie Skłodowska-Curie Actions Individual Fellowship (Proposal number 799973). AM is supported by a Fundação para a Ciência e a Tecnologia (FCT) PhD Fellowship (PD/BD141576/2018). Research in SL’s laboratory is supported by the Champalimaud Foundation, an European Research Council (ERC) Consolidator Grant (772827), and the FCT (PTDC/NEUSCC/4786/2014).

## Conflict of Interest

The authors declare that the research was conducted in the absence of any commercial or financial relationships that could be construed as a potential conflict of interest.

## Publisher’s Note

All claims expressed in this article are solely those of the authors and do not necessarily represent those of their affiliated organizations, or those of the publisher, the editors and the reviewers. Any product that may be evaluated in this article, or claim that may be made by its manufacturer, is not guaranteed or endorsed by the publisher.
